# Deterministic Diffusion Fiber Tracking Improved by Quantitative Anisotropy

**DOI:** 10.1371/journal.pone.0080713

**Published:** 2013-11-15

**Authors:** Fang-Cheng Yeh, Timothy D. Verstynen, Yibao Wang, Juan C. Fernández-Miranda, Wen-Yih Isaac Tseng

**Affiliations:** 1 Department of Biomedical Engineering, Carnegie Mellon University, Pittsburgh, Pennsylvania, United States of America; 2 Department of Psychology, Carnegie Mellon University, Pittsburgh, Pennsylvania, United States of America; 3 Department of Neurological Surgery, University of Pittsburgh School of Medicine, University of Pittsburgh Medical Center, Pittsburgh, Pennsylvania, United States of America; 4 Center for Optoelectronic Biomedicine, National Taiwan University College of Medicine, Taipei, Taiwan; 5 Department of Medical Imaging, National Taiwan University Hospital, Taipei, Taiwan; University of Maryland, College Park, United States of America

## Abstract

Diffusion MRI tractography has emerged as a useful and popular tool for mapping connections between brain regions. In this study, we examined the performance of quantitative anisotropy (QA) in facilitating deterministic fiber tracking. Two phantom studies were conducted. The first phantom study examined the susceptibility of fractional anisotropy (FA), generalized factional anisotropy (GFA), and QA to various partial volume effects. The second phantom study examined the spatial resolution of the FA-aided, GFA-aided, and QA-aided tractographies. An *in vivo* study was conducted to track the arcuate fasciculus, and two neurosurgeons blind to the acquisition and analysis settings were invited to identify false tracks. The performance of QA in assisting fiber tracking was compared with FA, GFA, and anatomical information from T_1_-weighted images. Our first phantom study showed that QA is less sensitive to the partial volume effects of crossing fibers and free water, suggesting that it is a robust index. The second phantom study showed that the QA-aided tractography has better resolution than the FA-aided and GFA-aided tractography. Our *in vivo* study further showed that the QA-aided tractography outperforms the FA-aided, GFA-aided, and anatomy-aided tractographies. In the shell scheme (HARDI), the FA-aided, GFA-aided, and anatomy-aided tractographies have 30.7%, 32.6%, and 24.45% of the false tracks, respectively, while the QA-aided tractography has 16.2%. In the grid scheme (DSI), the FA-aided, GFA-aided, and anatomy-aided tractographies have 12.3%, 9.0%, and 10.93% of the false tracks, respectively, while the QA-aided tractography has 4.43%. The QA-aided deterministic fiber tracking may assist fiber tracking studies and facilitate the advancement of human connectomics.

## Introduction

Diffusion MRI tractography is a useful tool for studying white matter pathways [1,2]. This unique technique for revealing physical brain connections can be used to define structural connectivity and map the human connectome [3,4], a topic that has gained considerable attention recently [5–8]. Based on the fiber orientations obtained from diffusion tensor imaging (DTI)[9], the trajectories of a fiber pathway can be tracked using deterministic fiber tracking methods, including the FACT (fiber assignment by continuous tracking) method [10] and the streamline tracking algorithm [11]. To further resolve crossing fibers, high angular resolution diffusion imaging (HARDI)[12] and diffusion spectrum imaging (DSI)[13] have been proposed to obtain diffusion orientation distribution functions (ODFs) for multiple fiber resolving. The adjuvant deterministic tracking approaches have been proposed to make use of multiple fiber orientations [14,15] and to obtain the connectivity matrix for human connectome studies [5,16–18]. However, to determine when the fiber tracking should be terminated, a lot of fiber tracking methods still use voxel-based index, such as fractional anisotropy (FA), generalized fractional anisotropy (GFA)[19], and anatomical information from T_1_-weighted images [20,21]. These voxel-based approaches cannot selectively remove noisy fibers because all fiber orientations in the same voxel share the same anisotropy value. Moreover, the voxel-based index such as FA and GFA has been shown to be susceptible to various sources of the partial volume effects [22–28]. Specifically, the anisotropy values can be affected by the partial occupation of crossing fibers, free water in ventricles, or gray matter; consequently, the fiber tracking methods based on FA and GFA may suffer from premature termination or false continuation. One solution to the noisy fiber and partial volume problems is using ODF-based index scaled with spin density information. The ODF-based index has been used by the deterministic fiber tracking implemented in MRtrix (http://www.nitrc.org/projects/mrtrix), DSI Studio (http://dsi-studio.labsolver.org), and Dipy (http://nipy.org/dipy). Nonetheless, this improvement has not yet been rigorously examined. Although several tractography evaluation studies have been conducted [29–31], they examined the overall tracking results, which can also be affected by the applied reconstruction methods and tractography algorithms. A study specifically comparing voxel-based and ODF-based index is yet to be conducted.

**Figure 1:**
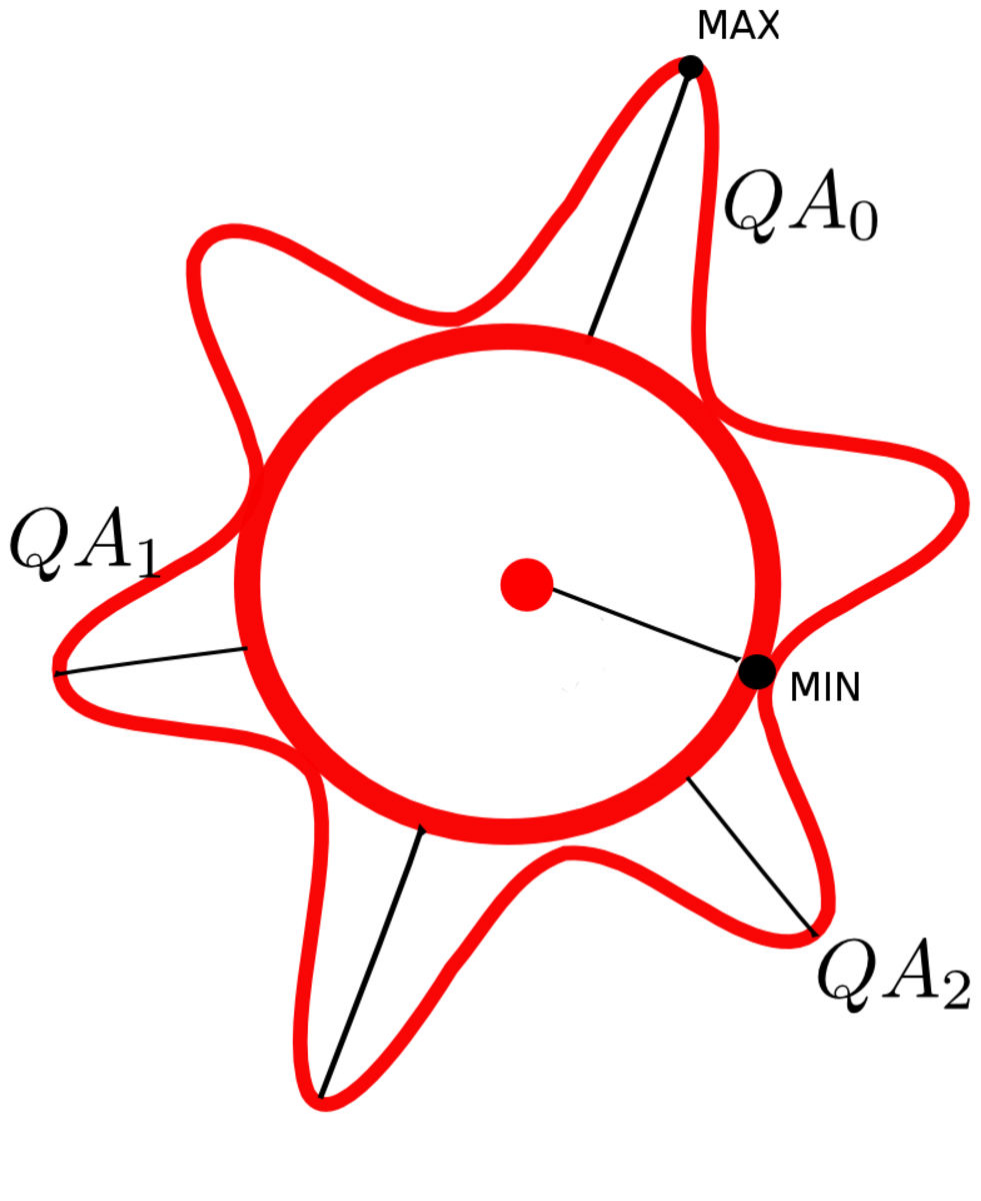
Diagram showing QA calculation from a spin distribution function. The red outer contour represents the spin distribution function calculated by generalized q-sampling imaging, whereas the sphere at the center is the isotropic component estimated by its minimum value. A QA value is defined for each peak orientation, and it serves as an index to differentiate less salient peaks and to selectively remove them. (Eleftherios Garyfallidis, "Towards an accurate brain tractography", PhD thesis, University of Cambridge, 2012, use with permission).

In this study, we conducted two phantom studies and an *in vivo* study to examine an ODF-based index called quantitative anisotropy (QA). QA is defined as the amount of anisotropic spins that diffuse along the fiber orientation [32]:

$$\text{QA}=Z_{0}\left(\psi \left(\hat{a}\right)-iso\left(\psi \right)\right)$$

(1)

where *ψ* is the spin distribution function (SDF) estimated using generalized q-sampling imaging [32]. $\hat{a}$is the orientation of the fiber of interest, and *iso*(*ψ*) is the isotropic background diffusion of the SDF. Z_0_ is a scaling constant that scales free water diffusion to 1 and makes QA value comparable across subjects. As shown in Figure 1, QA is defined for each peak orientation on an SDF, and it can be used to filter out false peaks. The difference between QA and regular ODF amplitude is that QA scales with spin density and the isotropic component is discarded, whereas regular ODF does not scale with spin density and is often min-max scaled to 0-1.

The first phantom study evaluated QA under different partial volume conditions, and the results were compared with those using FA and GFA. The performance was judged by examining which index was less sensitive to the partial volume effect and remained constant along the fiber pathway until its termination. The second phantom study used a fiber-wound phantom spindle to examine the FA-aided, GFA-aided, and QA aided tractography. The performance was judged by examining which approach can best reveal the geometry of the phantom fibers. The *in vivo* study evaluated the performance of the QA-aided tractography by tracking the arcuate fasciculus, and the results were compared with the FA-aided, GFA-aided, and anatomy-aided tractographies. To achieve a fair comparison, two neurosurgeons blind to the acquisition and analysis settings were invited to evaluate the tractography and to identify false trajectories. The performance was then reported by the percentage of false trajectories generated in different settings. 

## Materials and Methods

### Deterministic fiber tracking using quantitative anisotropy

**Figure 2:**
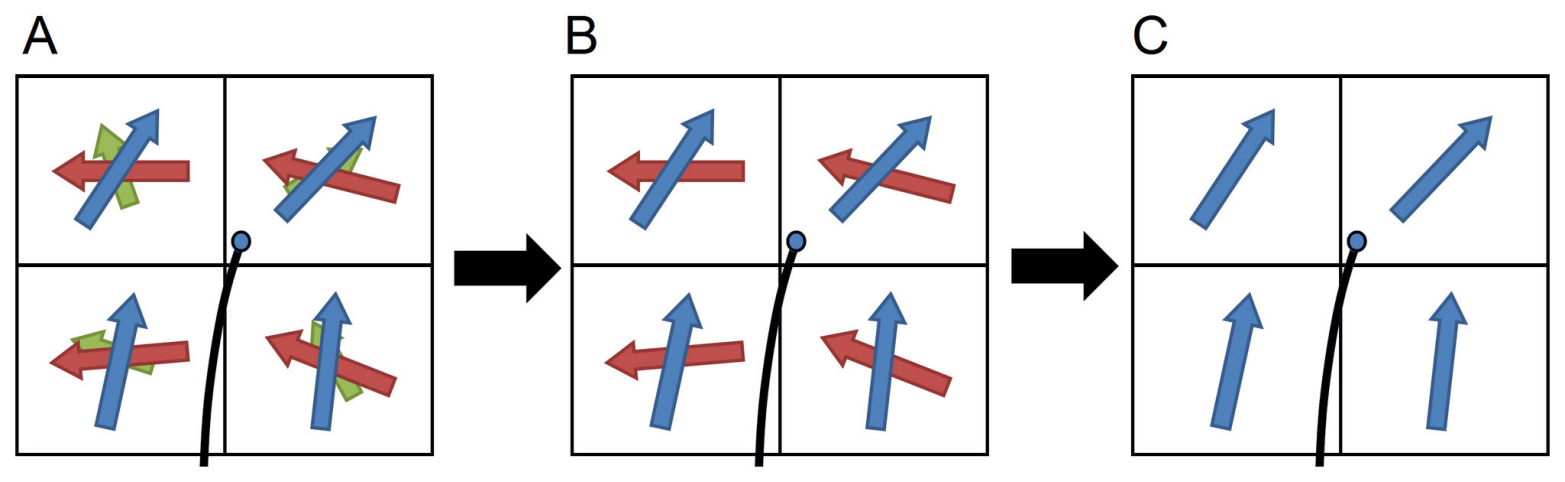
The filtering-selection process used in the generalized deterministic fiber tracking algorithm. (A) Each voxel may resolve multiple fiber orientations. (B) The noisy fibers are filtered using a pre-defined QA threshold. (C) The fiber orientations forming the least turning with respect to current propagation direction are selected.

Deterministic fiber tracking can be viewed as solving an ordinary differential equation [11], in which the fiber trajectory can be parameterized as **r**(*s*), **r**:R→R^3^, where *s* is the length of the trajectory, and **r**(*s*) is the trajectory in the brain. The fiber trajectory can then be calculated by Euler’s method:

$$r\left(s+\Delta s\right)\approx r\left(s\right)+\Delta s\cdot \hat{u}\left(r\left(s\right)\right)$$ (2)

where *Δs* is the step size of each propagation, and $\hat{u}\left(r\right)$ is the propagation direction estimated at coordinate **r**. The estimation of the propagation directions, $\hat{u}\left(r\right)$, faces two challenges. The first challenge is that each voxel may have multiple fiber orientations, and thus a strategy is needed to select the fiber orientation that is relevant to the trajectory. The second challenge is that the fiber orientations are resolved at discrete points, whereas the estimation of $\hat{u}\left(r\right)$ can occur at any coordinate. An interpolation approach is needed to bridge the gap between discrete data and continuous estimation. To address these two challenges, we used a filtering-selection approach as shown in Figure 2. The first stage filters out noisy fibers that have QA values lower than a pre-defined threshold (noisy fibers as annotated by green). The purpose of this filtering is to eliminate false fibers and to define the termination locations simultaneously. The second stage selects a fiber orientation for each voxel, and the selected orientation needs to satisfy two requirements. First, if a voxel has multiple fibers, the one that forms the smallest turning angle is selected. Second, the turning angle of the fiber orientation has to be less than a predefined angular threshold. The angular threshold is defined according to a priori knowledge of the trajectory curvature. Examples of selected fiber orientations are shown by blue arrows in Figure 2. After this filtering-selection process, the propagation direction is calculated using the following equation:

$$\hat{u}\left(r\right)=Z\sum_{R\in N\left(r\right)}w\left(r-R\right)\hat{v}\left(R\right)$$ (3)

where *Z* is a constant that normalizes the propagation direction. *N*(r) is a set of voxels that had selected fiber orientations around coordinate **r**. In a 3-dimensional setting, *N*(r) can contain up to 8 nearby voxels. For example, if **r** = (32.3, 12.4, 16.9), the nearby voxels include (12–12–12–12,13–13–13,16–16–16–16,17–17–17,32–32–32–32,33–33–33), and (13,17,33). One should note that some of the voxels may not reveal any fibers after the filtering-selection process; thus, the number of voxels in *N*(r) can be less than 8. *w* is the weighting function that gives higher weights to voxels closer to **r**. Here, trilinear interpolation is used, and the weighting function is defined as w(Δr)=, where$\Delta r_{x}$, $\Delta r_{y}$, and $\Delta r_{z}$ are the *x, y*, and *z* components ofΔr, respectively (Δris equal to r - R). is the fiber orientation obtained by our filtering-selection process applied to voxel **R**. The summation in Eq. (3) calculates the weighted sum of the selected fiber orientations, i.e.,$\hat{u}\left(r\right)$. The calculated fiber orientation at **r** is then used in Eq. (2) to conduct track propagation. The propagation continues until the sum of the weighting is less than 0.5, i.e., 

$$\sum_{R\in N\left(r\right)}w\left(r-R\right)<0.5$$ (4)

Details of the algorithms are listed in Table 1. The source codes are available on the DSI Studio website (http://dsi-studio.labsolver.org).

**Table 1:** Generalized Deterministic Tracking Algorithm Using Quantitative Anisotropy.

**Input**	**Comments**
Dir	The fiber orientations, e.g. dir[R] is a set of fiber orientations resolved at coordinate **R**
Qa	The fiber QA values, e.g. qa[R][$\hat{a}$] is the QA of the fiber at coordinate **R**, where is the fiber orientation.
**R**	Seeding point
$\hat{u}$	Initial propagation direction1
*θ_a_*	Angular threshold
*θ_q_*	QA threshold
*Δs*	Step size
**Output**	**Comments**
**T**	The coordinate list for the fiber trajectory
**Algorithm**	**Comments**
**1.u^'** ← 0, *w*← 0	u^'is the next propagation direction, and w is the accumulated weighting.
2. **T**←{**T**, r}	Record the current coordinate **r** in the trajectory list **T**
3. Repeat steps 4, 5, and 6 for all voxels **R** near **r**	Iterates 8 nearby voxels **R** at the location **r**
4.$\hat{v}$ ← 0	
5. for all fibers $\hat{a}$ in dir[R], if qa[R][$\hat{a}$] > *θ*_*q*_ and $\left|\langle \hat{u},\hat{a}\rangle \right|>\cos\left(\theta_{a}\right)$ and$\left|\langle \hat{u},\hat{a}\rangle \right|>\left|\langle \hat{u},\hat{v}\rangle \right|$ then $\hat{v}$←$\delta \left(\hat{u},\hat{a}\right)\hat{a}$	Iterate all fibers orientations $\hat{a}$ at **R** and select a fiber with QA greater than the QA threshold and has the smallest turning angle less than the angular threshold. =1 if $\langle \hat{u},\hat{a}\rangle$≥0 and -1 otherwise.
6. if $\hat{v}$ is not 0 then *w*←*w* + *f*(r - R) and u^'←+ *f*(r - R)$\hat{v}$	Accumulate the weightings using the weighting function f(Δr) ^[2^] and calculate the weighted sum of the fiber orientations
7. terminate algorithm if *w* < 0.5	Check the termination criterion
8.$\hat{u}$ ←u^'/|u^'|	Normalize the propagation direction.
9. r ← r +*Δs$\hat{u}$*	Propagate to the next location
10. repeat the algorithm from step 1	Repeat the algorithm
^[1]^ The tracking algorithm conducts tracking at one direction. To obtain the trajectory at the opposite direction, conduct the tracking algorithm again with initial directions of -$\hat{u}$.^[2^]^$f\left(\Delta r\right)=\left(1-\left|\Delta r_{x}\right|\right)\left(1-\left|\Delta r_{y}\right|\right)\left(1-\left|\Delta r_{z}\right|\right)$^, where$\Delta r_{x}$, $\Delta r_{y}$, and $\Delta r_{z}$ are the *x, y*, and *z* component of the displacement, respectively.	

One should note that if this algorithm is applied to DTI data and QA is replaced by FA, the algorithm will give a result similar to that of the streamline fiber tracking algorithm. Moreover, using the nearest neighbor as the weighting function (i.e., w(Δr)=1if Δr is the minimum, and w(Δr)=0 otherwise) and using an infinitesimally small step size will make this algorithm identical to the FACT algorithm. These features suggest that our generalized deterministic fiber tracking algorithm is actually a more general version of the deterministic tracking algorithm. These features also allow us to conduct a performance comparison by simply replacing QA with FA, GFA, or anatomical label.

### Phantom study

**Figure 3:**
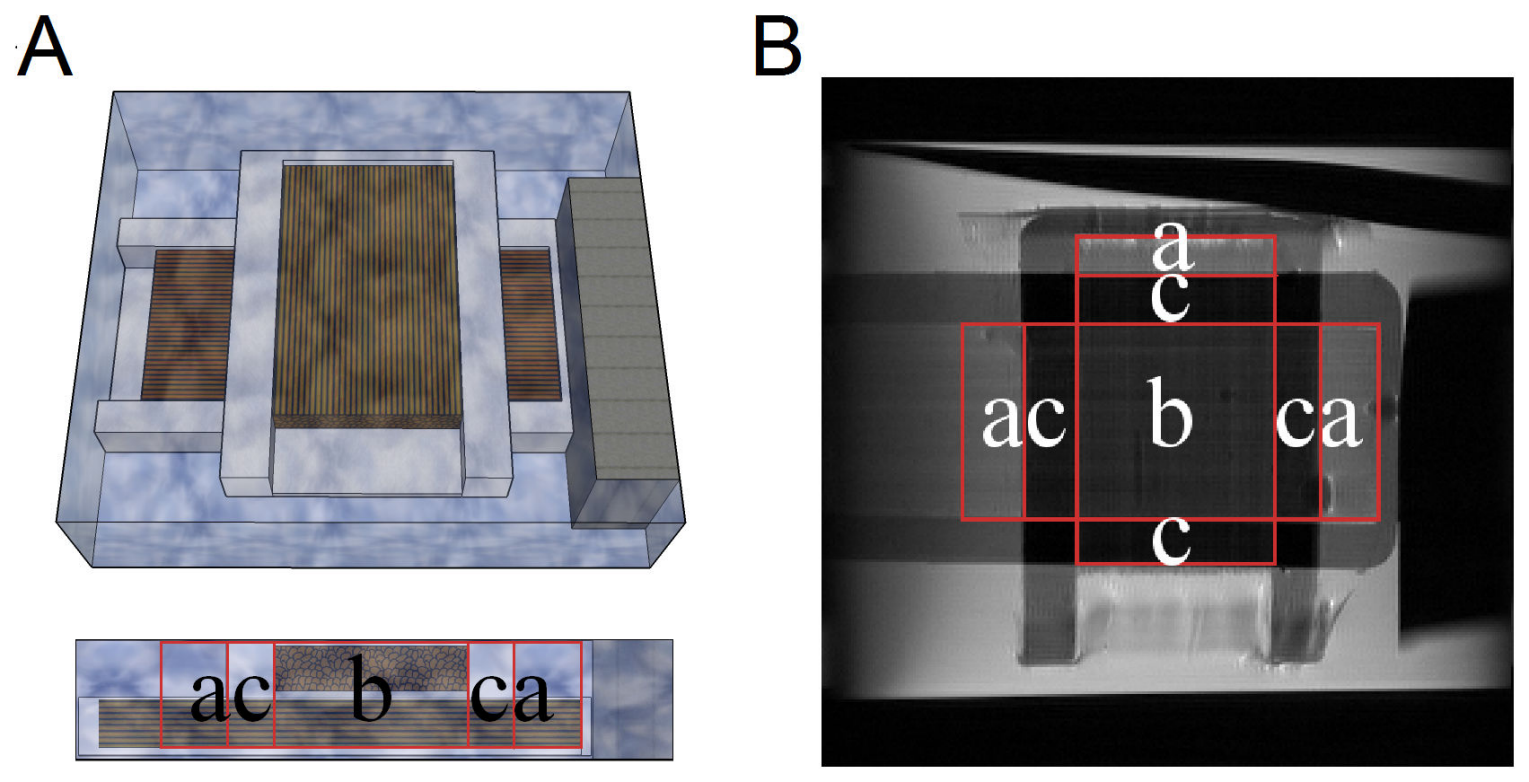
Layout of the diffusion phantom that simulates different partial volume conditions. (A) The layout of the phantom is presented by a 3D illustration and a lateral profile. (B) The layout of the phantom is presented with a T_2_-weighted image. The diffusion phantom examines three types of the partial volume effect: (a) fibers/free water, (b) fibers crossing, and (c) fibers/non-diffusive materials.

We simulated three partial volume conditions to examine the performance of QA compared with FA. A better index should be less susceptible to partial volume conditions and more constant along a fiber track until its termination. The diffusion phantom was constructed of silica capillary tubes (inner diameter of 20 μm and outer diameter of 90 μm; Polymicro Technologies, Phoenix, Arizona, USA) aligned in two placeholders stacked on one another. One placeholder was placed vertically, and the other was placed horizontally, forming a 90-degree crossing. The whole phantom was immersed in water, as shown in Figure 3A, where the layout was confirmed by a T_2_-weighted image, as shown in Figure 3B. As shown in the figures, the phantom layout simulated three partial volume conditions: (a) fibers/free water, (b) fibers crossing, and (c) fibers/non-diffusive materials. The phantom was scanned on a 9.4-Tesla Bruker spectrometer (Bruker Companies, Ettlingen, Germany). High angular resolution diffusion-weighted images (160 diffusion encoding directions in addition to the b0 image) were acquired using a 2D-FT stimulated-echo sequence with TR/TE = 1900/13.8 ms, matrix size = 32 × 32, FOV = 25 mm × 25 mm, slice thickness = 3.6 mm, diffusion time = 100 ms, diffusion gradient duration = 3 ms, b-value = 4000 s/mm^2^, and the average number of repetitions = 4. FA was calculated using diffusion tensor imaging. GFA was calculated using the ODFs reconstructed from q-ball imaging [33]. QA was calculated using generalized q-sampling imaging with a diffusion sampling ratio of 1.25, a recommended value according to the original study [32]. The distributions of FA, GFA, and QA under different conditions of the partial volume effects were separately plotted to examine the influence of the partial volume effects.

**Figure 4:**
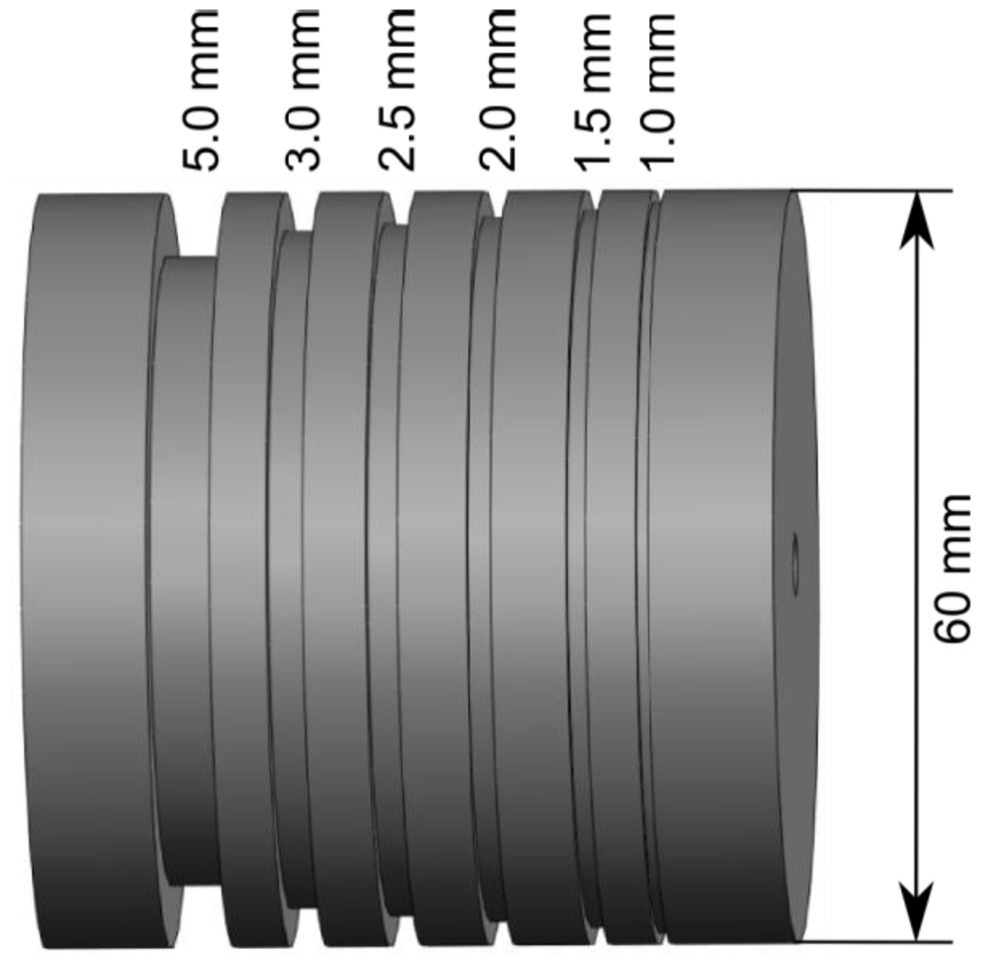
The design of a phantom spindle. The phantom has six different sized grooves wound with polyester fibers to examine the spatial resolution of a fiber tracking algorithm. (Bach Micheal et al., "DWI-Phantom–Resolution Phantom", http://www.nitrc.org/projects/diffusion-data , use with permission).

The second phantom study used a phantom spindle [34] to examine the spatial resolution of the FA-aided, GFA-aided, and QA-aided tractographies. The phantom was a cylinder-shaped plastic spindle with six different sized grooves on the perimeter, as shown in Figure 4. The grooves had polyester fiber threads wound in it. The diameter of each polyester fiber was 15 µm. The whole phantom was embedded in agarose gel. Diffusion images were acquired using 3 T Magnetom Trio scanner (Siemens, Erlangen, Germany) using an 8 channel knee coil and a monopolar single-shot echo planar imaging sequence. The spatial resolution was 2.5 mm isotropic. The FOV was 160 mm. TR = 2900 ms, TE = 78 ms. The GRAPPA acceleration factor was 2. A total of 180 diffusion directions were obtained with b = 1000 s/mm^2^, and 20 b0 images were acquired. The image data are publicly available at www.nitrc.org. To obtain the FA-aided tractography, we reconstructed the image data using diffusion tensor imaging. The tractography was generated by an angular threshold of 60 degrees and a step size of 1.25 mm (half of the voxel size in one dimension). The seeds were placed at the fiber strands. The FA threshold was 0.2. The value was determined by measuring the FA value of the smallest fiber strand on the phantom. Tracks with length under 40 mm were discarded to eliminate fiber fragments. A total of 1,000 tracks were generated. To obtain the GFA-aided and QA-aided tractography, we reconstructed the image data using generalized q-sampling imaging with a length ratio of 1.25 [32]. GFA was calculated using the ODFs reconstructed from q-ball imaging [33]. The GFA-aided and QA-aided tractography were obtained using the same tracking parameters except that the GFA threshold was 0.05 and that QA threshold was 0.04. The values were determined by measuring the GFA and QA values of the smallest fiber strand on the phantom. 

### In vivo diffusion MRI acquisition

A 25-year-old subject was scanned using a 3-T MRI system (TIM Trio; Siemens, Erlangen, Germany). Written inform consent was obtained from the subject, and our research procedures were approved by the Institutional Review Board of National Taiwan University Hospital. The scan was performed on a 12-channel coil, using a single-shot twice-refocused echo planar imaging (EPI) diffusion pulse sequence. The diffusion sampling schemes included the HARDI-253 (shell scheme) and DSI-203 (grid scheme) schemes that were previously documented in an optimization study [35]. The HARDI-253 scheme acquired 253 sampling points in the q-space, whereas the DSI-203 scheme acquired 203 sampling points. For the HARDI-253 scheme, the diffusion parameters were TR/TE = 7200 ms/133 ms, b-value = 4,000 s/mm^2^, and the scanning time was approximately 30 minutes. For the DSI-203 scheme, the diffusion parameters were TR/TE = 7200 ms/144 ms, maximum b-value = 4,000 s/mm^2^, and the scanning time was approximately 25 minutes. The shell and grid sampling schemes were acquired using the same spatial parameters: the field of view was 240 mm × 240 mm, the matrix size was 96 × 96, the slice thickness was 2.5 mm (no gap), and the number of the slices was 40 to cover the cerebral cortex, resulting in a voxel size of 2.5 mm × 2.5 mm × 2.5 mm. The generalized q-sampling imaging reconstruction was applied to both sampling schemes with a diffusion sampling length ratio of 1.25 as recommended in the original study. The ODF dimension of 642 was used, achieving an ODF angular resolution of 9 degrees. For the performance comparison, the diffusion tensor imaging and FA values were also calculated by DSI Studio. GFA was calculated using the ODF from q-ball imaging [33].

The QA-aided tractography was generated using QA as the termination index, whereas the FA-aided and GFA-aided tractographies were generated using FA and GFA as the termination indices, respectively. The anatomy-aided tractography used a white matter segmentation map, which was obtained by segmenting T_1_-weighted images using SPM8 (Wellcome Trust Centre for Neuroimaging, UK) with default parameters. To determine the comparable thresholds for FA, GFA, and QA, we plotted masks using different threshold levels and compared them with the white matter map obtained from SPM segmentation. The optimal values were determined by the mask that offers the best white matter coverage. An FA threshold of 0.12 was used in the FA-aided tractography, and this value agreed with the recommended value of approximately 0.1 [36]. The comparable GFA threshold was 0.055, whereas the comparable QA threshold was 0.63. In the anatomy-aided tractography, values greater than zero in the white matter map represented white matter regions, and fiber orientations in non-white-matter region (e.g. gray matter, CSF…etc.) were discarded. One should note that the FA, GFA, and QA values can be affected by b-value and diffusion sampling scheme, and thus the optimal value should be determined by comparing these anisotropy values with the white matter mask.

The tractography of the arcuate fasciculus was generated for performance comparison. A spherical region of interest (ROI), 14 mm in radius, was placed at the temporal-parietal junction. Fibers passing the ROI and connecting temporal with frontal regions were selected. The ROI placement and track selection were conducted using the interface provided by DSI Studio (http://dsi-studio.labsolver.org). The tractography was generated by an angular threshold of 60 degrees and a step size of 1.25 mm (half of the voxel size in one dimension). Whole-brain seeding was conducted until a total of 2,000 fiber tracks were generated from the ROI. We used a pseudorandom number generator to place the seeds, and the seed distribution was deterministically random. This ensured that the same seeding sequence was used. The same ROI and parameter settings were used to reconstruct the arcuate fasciculus in both the FA-aided, GFA-aided, anatomy-aided, and QA-aided tractographies for comparison. The same ROI and parameter settings were used to reconstruct the arcuate fasciculus in the FA-aided, GFA-aided, anatomy-aided, and QA-aided tractographies for comparison. 

We further compared the QA-aided tractography with the tractography obtained from constrained spherical deconvolution (CSD)[37]. The HARDI-253 dataset was analyzed using MRtrix (www.nitrc.org/projects/mrtrix‎). As suggested in the MRtrix’s user document, the response function was estimated using voxels with FA value greater than 0.7 and a maximum harmonic order of 6. The estimated response function was inspected to ensure its accuracy. The CSD was calculated using a recommended maximum harmonic order of 8. Deterministic fiber tracking was conducted using MRtrix’s deterministic fiber tracking routine. The same ROIs were supplied to generate the tractography of arcuate fasciculus with a total of 2,000 tracks.

### Ethics Statement

Data were analyzed anonymously, and written inform consent can be waived because the study fulfills the following requirements. The study has the lowest risk. The risk to the studied subjects does not exceed the possible risks of people who do not participate in the study. Waiving the prior consent does not affect the rights and interests of the studied subjects. (File# 1010265083, Ministry of Health, Executive Yuan, Taiwan). The research procedures were also approved by the Institutional Review Board of National Taiwan University Hospital.

### Blind evaluation by two neurosurgeons

The blind evaluation was performed independently by two neurosurgeons (Y. W. and J. C. F-M. in the Surgical Neuroanatomy Lab at the Department of Neurosurgery, University of Pittsburgh. The neurosurgeons had conducted extensive micro-dissection studies on formalin-fixed human brain specimens and had been familiarized with the anatomical structures of the arcuate fasciculus before the tractography examination. The tractography results were examined by the neurosurgeons to identify false tracks. The false tracks were picked manually using the track selection interface provided by DSI Studio. The neurosurgeons received no information regarding the acquisition schemes or tractography algorithms, and the examination results were graded by the average percentage of fiber tracks that were identified as false tracks. Because each fiber track in the tractography was generated independently, the 95% confidence intervals of the results were obtained by applying bootstrap resampling to the generated fiber tracks. One should note that this resampling did not consider the error due to the expert evaluation. The bootstrap resampling was conducted by randomly selecting a track with replacement from 2,000 tracks generated from the tracking algorithm. This random selection was repeated 2,000 times to constitute a sample of the fiber bundles. A total of 1,000 samples were obtained to calculate the 95% confidence intervals of percentage of the fiber tracks that were identified as false tracks.

### Evaluation using the cortical surface rendered from T_1_-weighted images

In additional to expert evaluation, T_1_-weighted images were used to conduct a qualitative comparison of the termination locations of the fiber tracks. The T_1_-weighted images were linearly registered to the DSI space by minimizing the mutual information. The T_1_-weighted images were then also segmented using SPM8 (Wellcome Trust Centre for Neuroimaging, UK), and the segmentation result was used to render the cortical surface. The tractography was overlapped by the rendered cortical surface to examine whether the termination locations of the fiber tracks were in good agreement with the gyral folding. The linear registration and cortical surface rendering were conducted using a built-in function provided by DSI Studio. The cortical surface was presented along with the fiber tracks to examine whether the termination locations of the tracks matched the gyral folding.

## Results

### Phantom study

**Figure 5:**
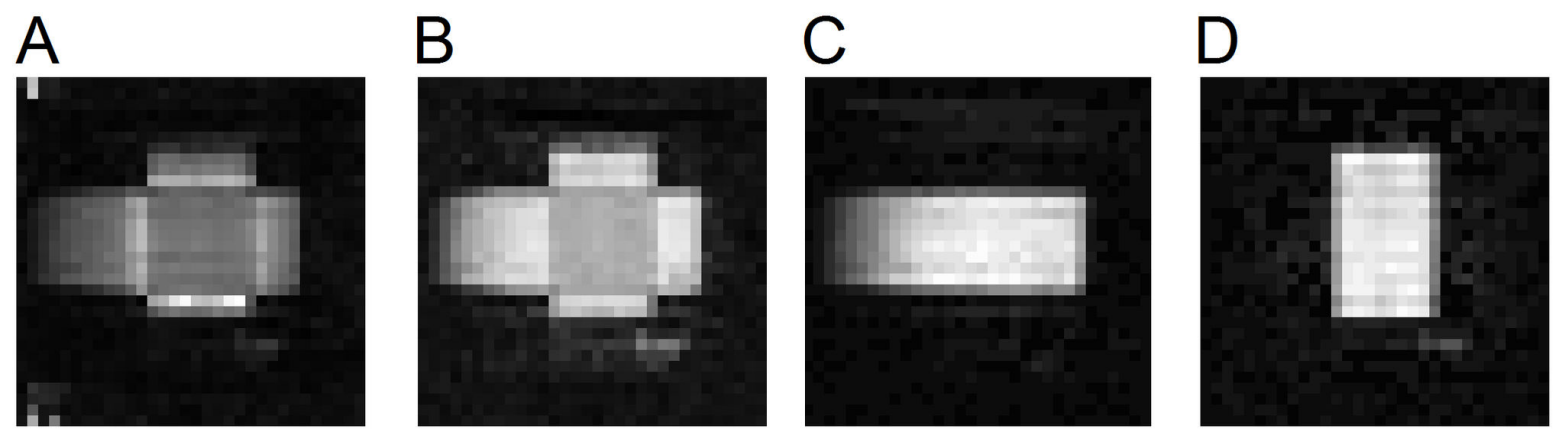
FA, GFA, and QA maps of the diffusion phantom. (A) The FA map shows inhomogeneous intensity across the fiber bundles. Decreased values of FA can be observed in partial volumes of crossing fibers and free water, whereas increased values can be observed in partial volumes of the non-diffusive materials. The susceptibility of FA to the partial volume effect may result in poor track terminations. (B) The GFA map also shows reduced intensity at the fiber crossing region. (C) The QA map of the horizontal fibers shows homogeneous values regardless of different partial volume conditions. (D) The QA map of the vertical fibers also shows homogeneous values, suggesting that QA may serve as a better index for QA filtering and track termination.

The FA map of the diffusion phantom is shown in Figure 5A, and the GFA map of the diffusion phantom is shown in Figure 5B. The QA maps of the horizontal and vertical fibers of the diffusion phantom are shown in Figure 5C and 5D, respectively. The intensity of each anisotropy map was scaled such that the maximum value was presented by the maximum intensity, i.e. 255. As shown in Figure 5A, the FA map presents inhomogeneous intensity across the phantom, and lower intensity can be observed in regions with partial volumes from crossing fibers or free water. Similarly, the GFA map shown in Figure 5B also presents lower intensity in crossing fiber regions. By contrast, the QA maps show relatively homogeneous intensity in both the horizontal (Figure 5C) and vertical (Figure 5D) fibers regardless of the partial volume conditions. The results suggest that QA is less sensitive to the partial volume effects and thus is a robust index for defining track termination. 

**Figure 6:**
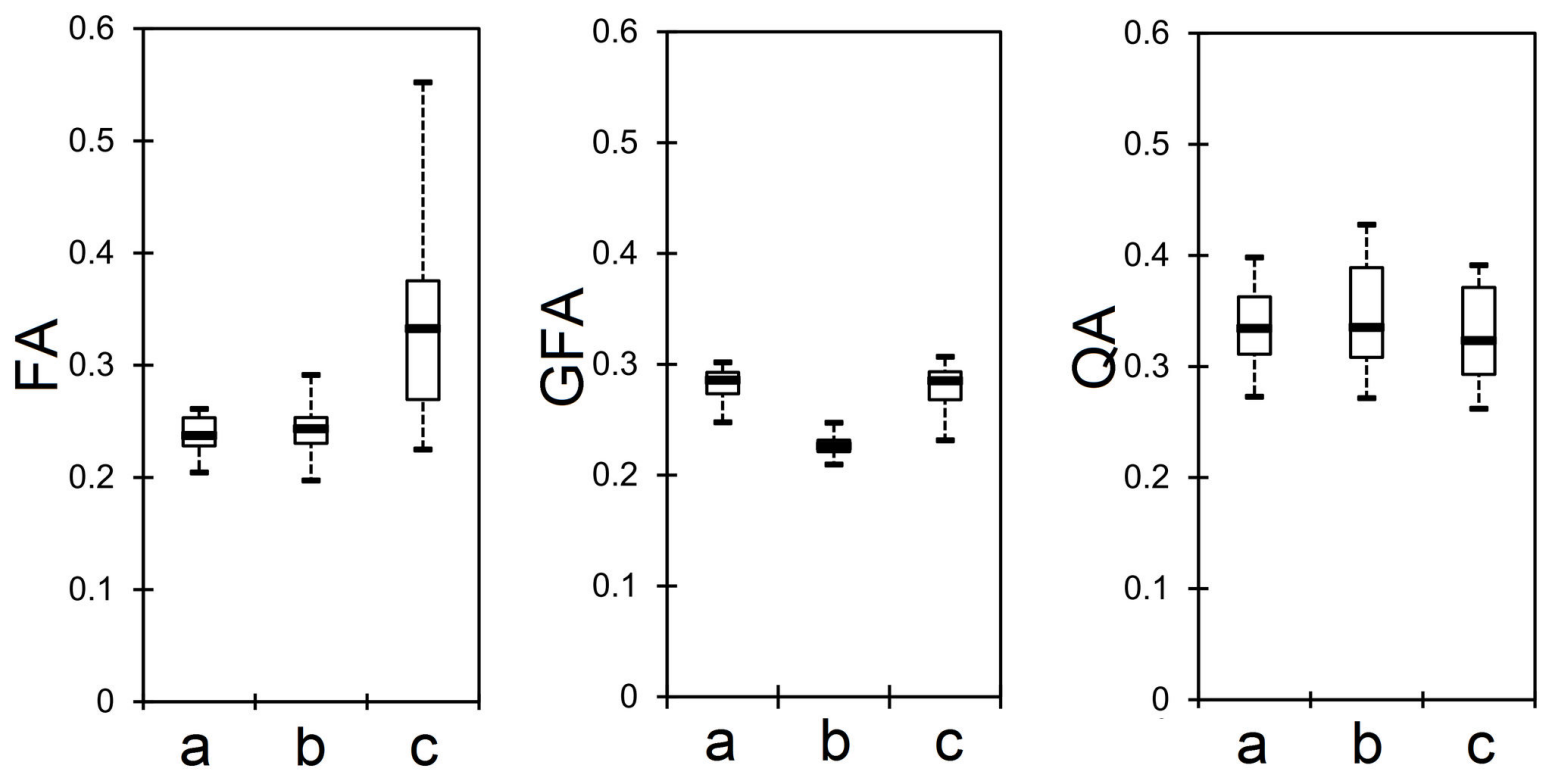
Box plots showing the first quartile, median value, and third quartile of FA, GFA, and QA distributions under three different partial volume conditions: (a) fibers/free water, (b) fibers crossing, and (c) fibers/non-diffusive materials. The FA and GFA are affected by the partial volume conditions, whereas the QA distribution remains relatively consistent regardless of the partial volume conditions.

The box plots in Figure 6 show the first quartiles, medians, and third quartiles of the FA, GFA, and QA distributions in the phantom study. The distributions were plotted against three types of partial volume conditions: (a) fibers/free water, (b) fibers crossing, and (c) fibers/non-diffusive materials. The FA distribution shows decreased values in the partial volumes from free water and crossing fibers, suggesting that using FA to determine track termination may be affected by the partial volume effect. The GFA distribution is less affected by the partial volume effect from free water, but it is still affected by the presence of crossing fibers. By contrast, the QA distribution shows a relatively consistent distribution regardless of the partial volume conditions, suggesting that QA is a quantitative measurement that is less affected by the partial volume effect and that it may serve as a better index to filter fiber orientations and to define track termination in deterministic tractography.

**Figure 7:**
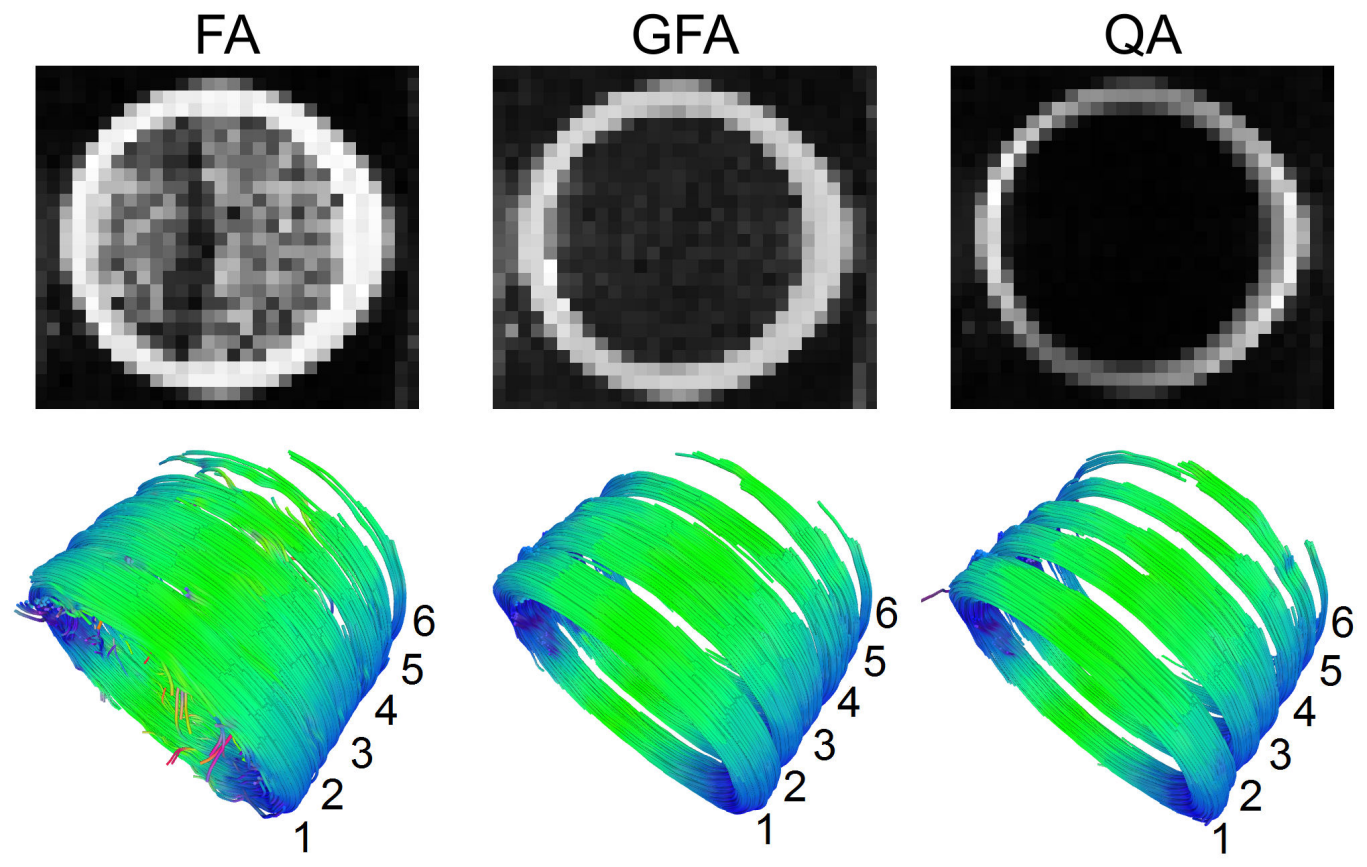
FA, GFA, and QA results of the phantom spindle. The first row shows the FA, GFA, and QA maps of the largest fiber strand wound around the phantom. The second row shows the FA-aided, GFA-aided, and QA-aided tractographies of the entire phantom spindle. The FA map shows prominent noise, whereas GFA and QA maps show less noise effect. The FA-aided tractography cannot resolve each individual fiber strand, and there are several false branching tracks. The GFA-aided tractography shows fewer false tracks, but the second and third strands bleed together, while a substantial part of the fifth strand is missing. The QA-aided tractography can resolve all strands, and most of the fifth strand remains visible.

Figure 7 shows the FA, GFA, and QA results of the phantom spindle. The first row shows the FA, GFA, and QA maps of the largest fiber strand that wound around the phantom spindle. These maps are min/max scaled to 0/255 to facilitate visualization. The FA map shows prominent noise at the center of the phantom, whereas the GFA map shows less noise, and the QA map shows the least. The noise may lead to erroneous trajectories and affect the accuracy of the fiber tracking algorithm. The second row shows the FA-aided, GFA-aided, and QA-aided tractographies of the entire resolution phantom. In each tractography, the first strand (largest) is placed at the bottom left-hand corner, and the other strands follow consecutively. The FA-aided tractography shows several false tracks branching from the center due to the noise. The first four strands bleed together and are not resolvable. This poor performance is due to the noise that corrupts the FA map and causes false continuation that bridges the neighboring fiber strands. The GFA-aided tractography shows fewer false branching tracks. However, the second and third strands bleed together, while a substantial part of the fifth strand is missing. This suggests that there is no optimal threshold that can retain the fifth strand (requires a lower threshold) and separate second and third strands (requires a high threshold) simultaneously. In contrast, the QA-aided tractography shows fewer false branching tracks than the FA-aided tractography. The second and third strands can be resolved, while most of the fifth strand remains visible. This result suggests that QA-aided tractography has better resolution power than FA-aided and GFA-aided tractography.

### In vivo study

**Figure 8:**
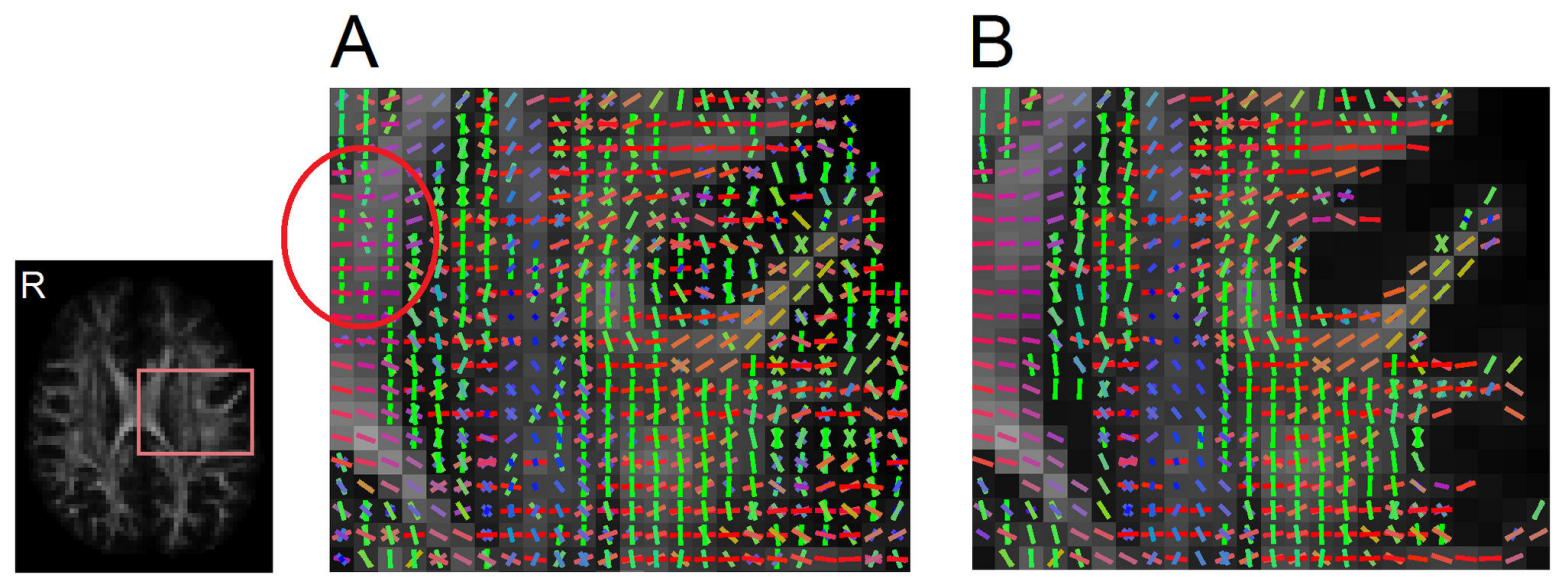
Fiber orientations before and after QA filtering. (A) Original fiber orientations without QA filtering. The noisy fibers that may lead to tracking errors can be observed in the white matter. (B) The fiber orientations filtered by the QA threshold shows clearer orientation mapping that can assist tracking the fiber trajectories.

The performance of the QA filtering is examined in Figure 8, where Figure 8A shows all the original fibers before QA filtering, and Figure 8B shows the remaining fibers after QA filtering. The figure is the axial view located near the centrum semiovale, a region where the corticospinal tract, corpus callosum, and arcuate/superior longitudinal fasciculus form a three-way crossing. The noisy fibers (green sticks in the annotated circle) in the corpus callosum (red sticks in the left half of Figure 8A) may cause false continuation or false termination in fiber tracking. In Figure 8B, the QA filtering eliminates the noisy fibers and simultaneously defines the termination locations (see the gray-white matter junction in Figure 8B) to facilitate deterministic fiber tracking. 

**Figure 9:**
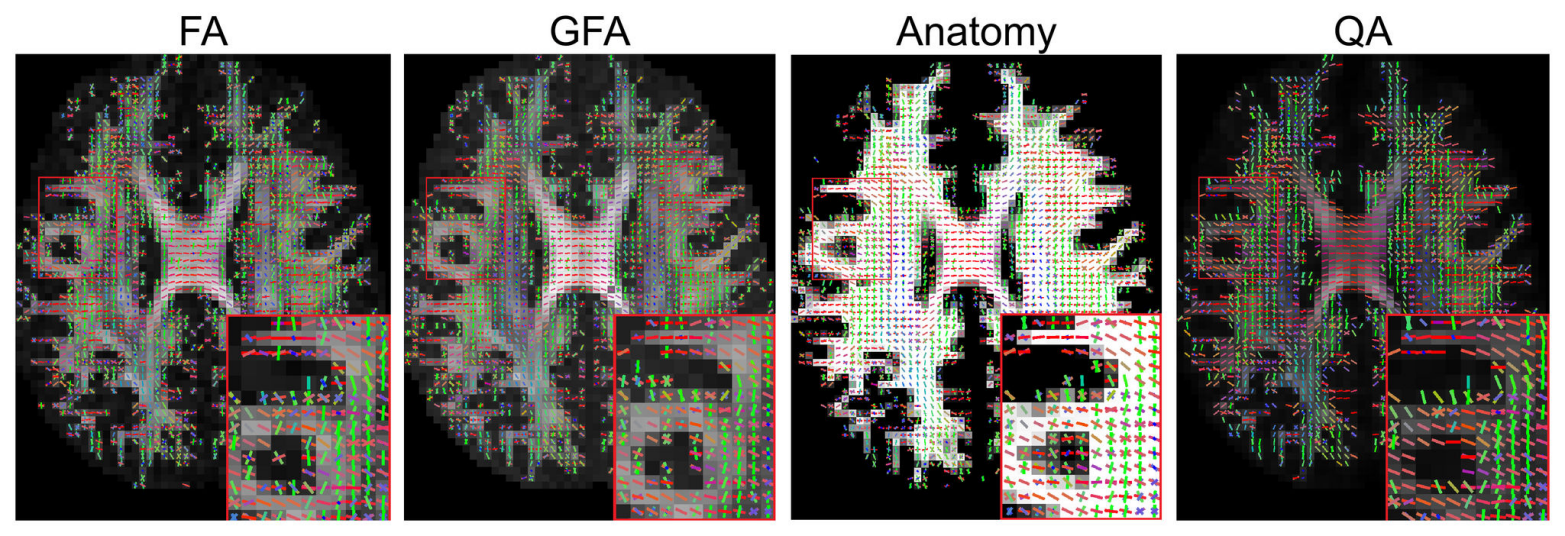
The filtering results using FA, GFA, anatomical information, and QA. FA, GFA, and anatomy filtering cannot eliminate noisy fibers in the white matter area because all the fiber orientations in a voxel share the same index value. The filtering result is susceptible to noisy fibers that may lead to false continuation or false termination. By contrast, QA filtering can eliminate noisy fibers and also define termination locations. Its clearer orientation map can assist the deterministic fiber tracking algorithm in defining the fiber pathways.

The QA filtering is further compared with FA, GFA, and anatomical filtering in Figure 9. Although the FA, GFA, and anatomical information can be used to define the termination location, it cannot eliminate noisy fibers, as shown in the inset figures. This limitation is due to the fact that all fiber orientations within the same voxel share the same FA or GFA value as well as the same anatomical information. Consequently, fiber tracking based on FA, GFA, and anatomical information may result in false turning due to these noisy fibers. By contrast, the inset figure of QA filtering shows clearer fiber orientation profile and less noisy fibers near the gray-white matter junction. The QA value is defined for each fiber orientation, a feature that enables deterministic fiber tracking to selectively eliminate noisy fibers and at the same time define accurate termination locations.

**Figure 10:**
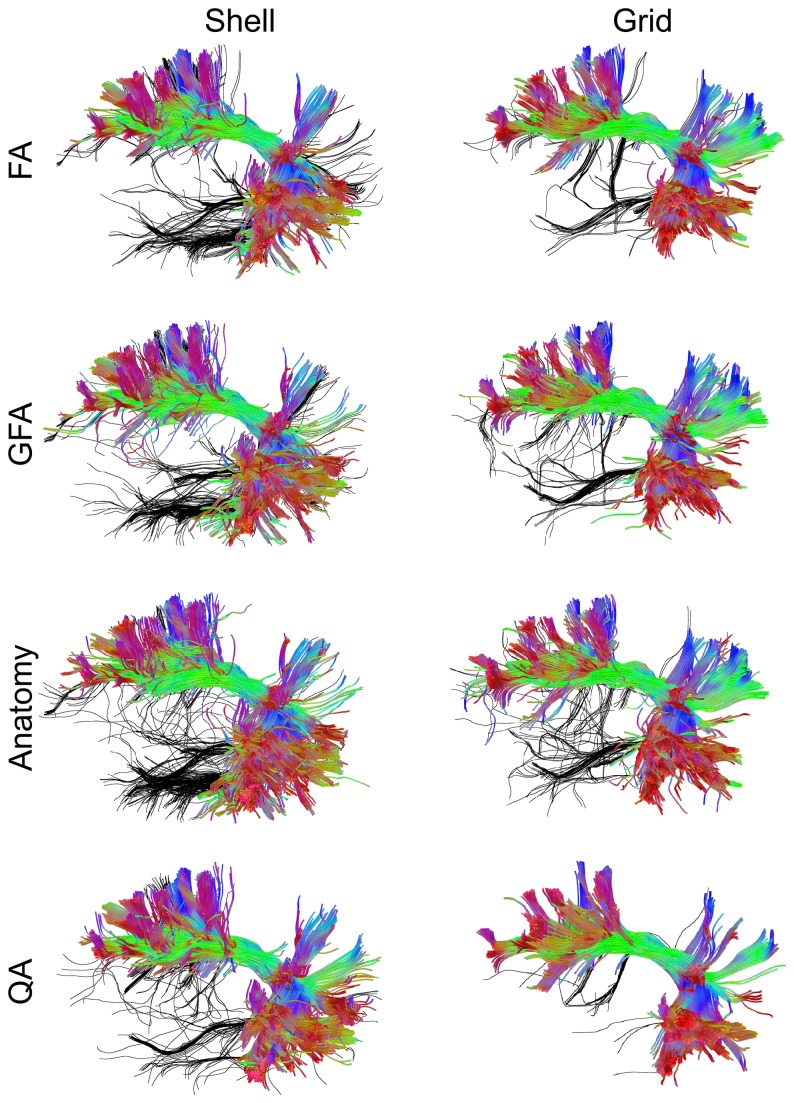
The performance of the FA-aided, GFA-aided, anatomy-aided, and QA-aided tractographies showing arcuate fasciculus using shell and grid sampling data. The false tracks identified by a neurosurgeon (Y. W.) are colored in black, whereas accurate trajectories are coded by directional color. The tractographies using FA, GFA, and anatomical information shows substantially more false tracks than that using QA. The best performance can be observed in the tractography using QA and the grid dataset.

Figure 10 shows the arcuate fasciculus reconstructed by the FA-aided, GFA-aided, anatomy-aided, and QA-aided tractographies. The same fiber tracking algorithm was applied to grid (DSI-203) and shell (HARDI-253) datasets. The false tracks identified by one of the neurosurgeons (Y. W.) are colored in black, whereas the remaining tracks are color coded by the local directions. The tractography generated using FA, GFA, and anatomical information show substantially more false tracks than those from QA. The false tracks branching from the temporal lobe may be due to false continuation of the noisy fibers that connect to the neighboring fiber pathways. It is noteworthy that the FA-aided, GFA-aided, and anatomy-aided tractographies show similar performance. This result can be expected because both FA and GFA can accurately separate white matter from gray matter, and the separation result is consistent with the white matter map as shown in Figure 9. This suggests that the anatomical information may have limited contribution to track termination because FA and GFA already provide similar information. The tractography aided by QA, by contrast, shows better performance, and the one using the grid dataset has the best performance with few errors. This outperformance can be attributed to the fact that QA can selectively remove noisy fibers, whereas FA, GFA, and anatomical information cannot achieve this because all fibers within the same voxels share the same index.

The evaluation results from each neurosurgeon (Y. W. and J. C. F.-M) are listed in Table 2. The results present good agreement between the two neurosurgeons: the QA-aided tractography outperforms the FA-aided, GFA-aided, and anatomy-aided tractographies regardless of the shell or grid sampling schemes

**Table 2:** Percentage of false tracks in tractography aided by different termination indices.

Scheme	Examiner	FA-aided	GFA-aided	Anatomy-aided	QA-aided
Shell	Y. W.	27.95%	30.2%	18.7%	12.5%
	J. C. F-M.	33.45%	35%	30.2%	19.9%
Grid	Y. W.	8.15%	8%	8.5%	1.85%
	J. C. F-M.	16.45%	10.05%	13.35%	7%

**Figure 11:**
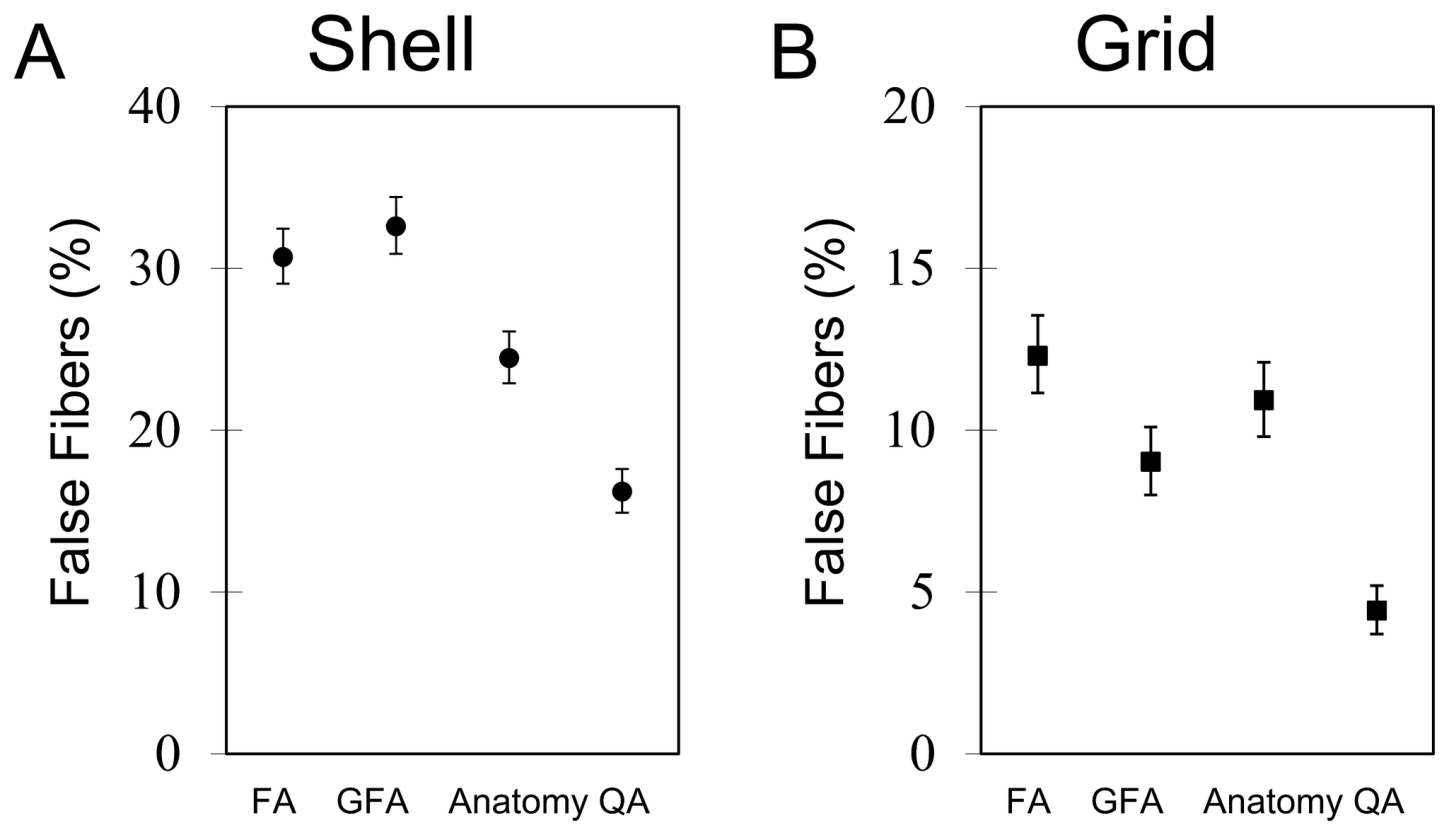
The average percentage of false tracks in the FA-aided, GFA-aided, anatomy-aided, and QA-aided tractographies generated from the (A) shell and (B) grid sampling schemes. The QA-aided tractography offers fewer false tracks than the FA-aided, GFA-aided, and anatomy-aided tractographies. The outperformance is achieved regardless of the diffusion sampling schemes.

Figure 11 shows the percentages of false tracks in the FA-aided, GFA-aided, anatomy-aided, and QA-aided tractographies averaged from the evaluation results of the neurosurgeons. The 95% confidence intervals calculated from bootstrap resampling are also shown in the figure. In the shell sampling scheme, the FA-aided, GFA-aided, and anatomy-aided tractographies show 30.7%, 32.6%, and 24.45% false tracks, respectively, whereas the QA-aided tractography shows 16.2% false tracks. In the grid sampling scheme, the FA-aided, GFA-aided, and anatomy-aided tractographies show 12.3%, 9.0%, and 10.93% false tracks, respectively, whereas the QA-aided tractography shows 4.43% false tracks. The QA-aided tractography outperforms the FA-aided, GFA-aided, and anatomy-aided tractographies regardless of the shell or grid sampling schemes

**Figure 12:**
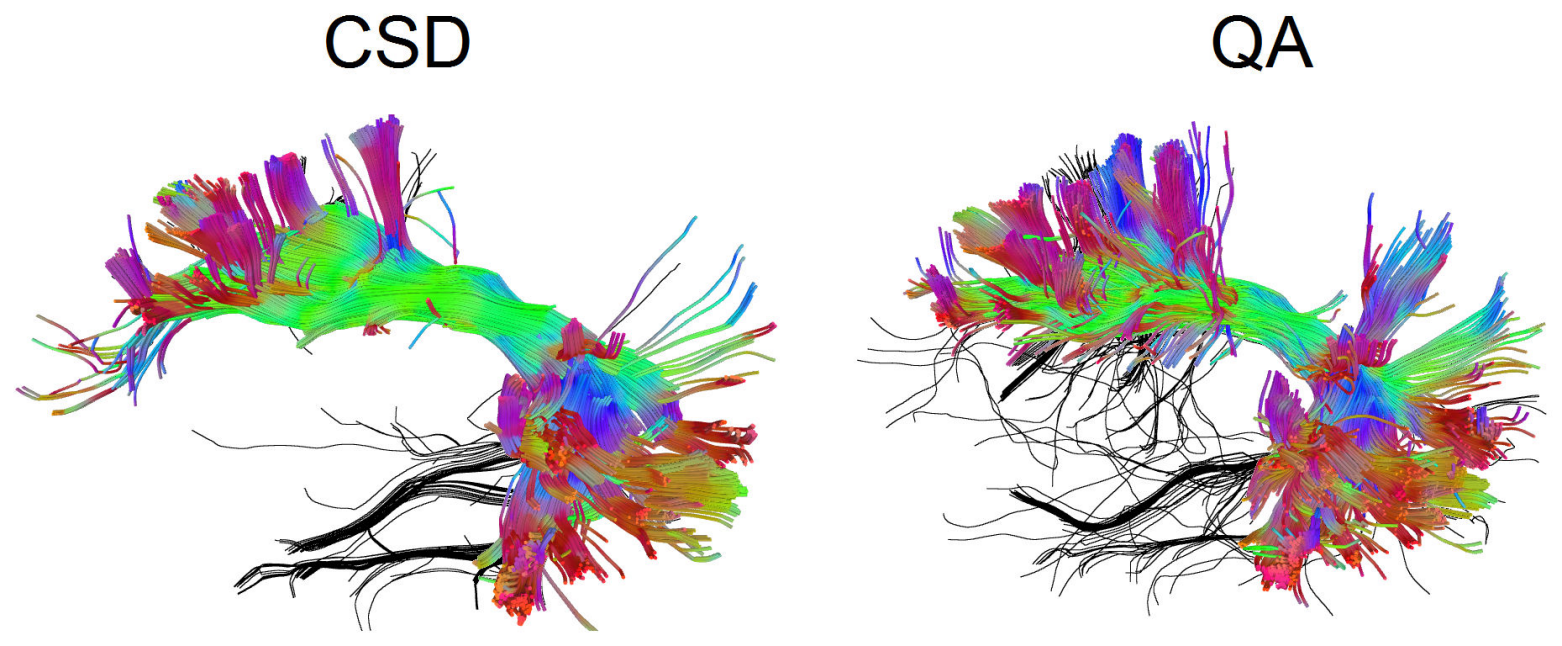
The QA-aided tractography compared with the CSD tractography. Both tractographies were generated using the same HARDI dataset. The QA-aided tractography used spin distribution functions to resolve crossing fibers without deconvolution, whereas CSD tractography used constrained spherical deconvolution to improve the angular resolution. The false tracks identified by a neurosurgeon (Y. W.) are colored in black, whereas accurate trajectories are coded by directional color. The QA-aided tractography shows an average of 16.20% of false fibers, whereas the CSD tractography shows an average of 15.38% fiber fibers. The difference is within the error range (1.3%) estimated using bootstrap resampling.

Figure 12 compares the QA-aided tractography with the CSD tractography. Both tractographies were generated using the same HARDI dataset. The CSD tractography used constrained spherical deconvolution to resolve crossing fibers, whereas the QA-aided tractography used SDFs to resolve crossing fibers without applying any ODF sharpening method. The false tracks identified by a neurosurgeon (Y. W.) are colored in black, whereas accurate trajectories are coded by directional color. The blind evaluation shows that the QA-aided tractography has 12.5% and 19.9% of false fibers identified by Y. W. and J. C. F.-M, respectively, and that the CSD tractography has 10.4% and 20.35% of false fibers identified by Y. W. and J. C. F.-M, respectively. In average, the QA tractography shows 16.20% false fibers, whereas the CSD tractography shows 15.38% false fibers. The difference is within the error range (1.3%) estimated using bootstrap resampling, suggesting that the QA-aided tractography based on SDFs has comparable performance as the CSD tractography. 

**Figure 13:**
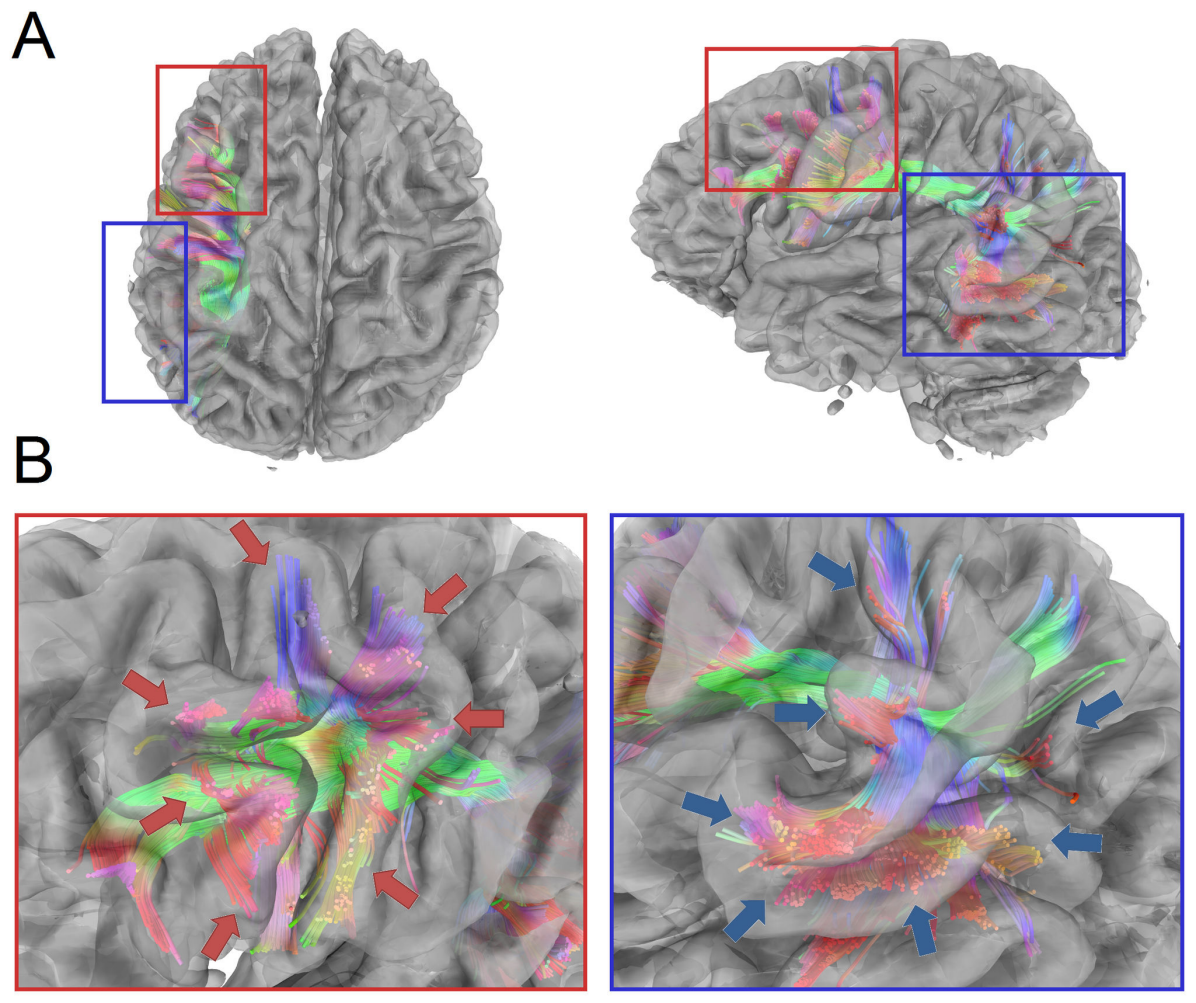
The QA-aided tractography of the arcuate fasciculus examined by the cortical surface rendered from T_1_-weighted images. (A) The axial (from the top) and coronal (from the left) views show the tractography of the arcuate fasciculus overlapped with the cortical surface. The red rectangle indicates the anterior termination location of the arcuate fasciculus, whereas the blue one indicates the posterior. (B) The zoom-in figures show that the termination locations of the fiber trajectories match well with the gyral folding (arrow) rendered from the T_1_-weighted images.

Figure 13 examines the termination locations of the QA-aided tractography (obtained from the grid dataset) of the arcuate fasciculus using the cortical surface rendered independently from T_1_-weighted images. Figure 13A presents the tractography in axial (from the top) and coronal (from the left) views overlaid with the cortical surface. The T_1_-weighted images have a much higher resolution (1 mm) and can serve as an external validation to check whether the terminations of the fibers are in good agreement with the gyral folding. The red rectangular region zooms in the anterior termination location of the QA-aided tractography, whereas the blue one zooms in the posterior termination location. The zoom-in figures in Figure 13B show that terminations of the fiber tracks (arrow) match well with the gyral folding, suggesting that QA can define good termination locations in deterministic fiber tracking.

**Figure 14:**
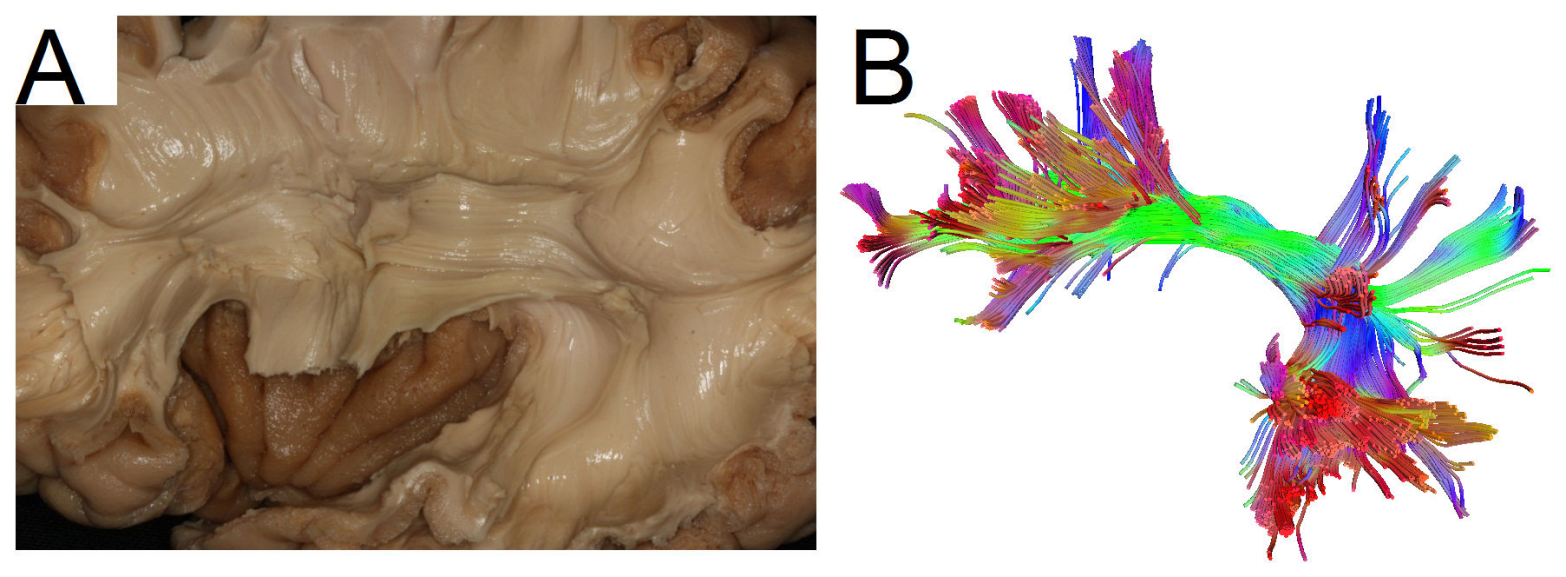
An example of arcuate fasciculus micro dissection and the QA-aided tractography. (A) The dissection of the arcuate fasciculus, pictured from the left hemisphere. (B) The tractography is generated by applying generalized deterministic fiber tracking to the grid sampling data with QA filtering. Although the images are from different subjects, remarkably consistent structures can be observed in both images, including the branching tracks to the prefrontal cortex and temporal gyrus.

Figure 14A shows micro dissection of the arcuate fasciculus viewed from the left hemisphere (courtesy of the Surgical Neuroanatomy Lab at the Department of Neurosurgery, University of Pittsburgh), and Figure 14B shows the arcuate fibers generated by the QA-aided deterministic fiber tracking using the grid dataset. Although Figures 14A and 14B were obtained from different subjects and by different operators, they show remarkably consistent anatomical features. The same branching fiber pathways connecting the inferior and middle frontal gyri with the inferior parietal lobule and superior and middle temporal gyri can be observed in both figures, suggesting that the QA-aided deterministic fiber tracking can be used as a tool to study the connectivity patterns of fiber tracks in the human brain.

## Discussion

We showed that deterministic fiber tracking can be improved by using QA to filter noisy fibers and define termination locations. Our first phantom study suggested that QA is less susceptible to the partial volume effects of free water, crossing fibers, and non-diffusive materials. The second phantom study also showed that QA has less noise, and the QA-aided tractography has better spatial resolution than the FA-aided and GFA-aided tractography. The *in vivo* experiment tested FA, GFA, anatomical information, and QA using the single-shell scheme (also known as HARDI) and grid scheme (also known as the DSI sampling scheme). The result indicates that the QA-aided tractography achieves the highest accuracy regardless of the applied sampling schemes. Further analysis showed that the QA-aided tractography based on SDFs has comparable performance as the CSD tractography. Although this QA-aided tractography has been adopted in DSI Studio to facilitate more than 20 tractography studies [38–44](complete list at http://dsi-studio.labsolver.org/publications), this paper is the first to conduct a systematic comparison to demonstrate its performance.

The outperformance of QA can be discussed in two aspects. The first aspect is that QA is less susceptible to the partial volume effect. This outperformance can be attributed to the fact that QA scales with the spin density, whereas FA does not. Incorporation of the spin density enables QA to quantify the amount of spins that diffuse in the fiber orientation, and this absolute amount of diffusive spins is not affected by adding additional partial volumes of free water. By contrast, FA or GFA, by definition, is a ratio that represents the overall anisotropy. Any additional isotropic component will reduce the FA value, resulting in the partial volume effect. One should note that incorporation of the spin density in QA presents other problems. QA can be affected by T_2_ shine-through effect, which can be corrected using b0 images acquired at different echo times. Although we did not demonstrate it in this study, the T_2_ effect on fiber tracking results can be neglected because the T_2_ relaxation time does not vary too much in the white matter. QA can also be affected by the signal inhomogeneity caused by B1 field inhomogeneity or non-uniform sensitivity of the receiving coils. To address this problem, further development of new pulse sequences or post-processing methods are required. The second aspect of the outperformance can be attributed to QA filtering. If multiple fibers are resolved within a voxel, QA can offer an index for each resolved fiber orientation, and the noisy fibers can thus be filtered out by QA. This feature is noteworthy because anatomical information from T_1_-weighted images can also facilitate track termination [20], but they cannot selectively remove noisy fibers within the same voxel. By contrast, QA filter can remove noisy fibers and prevent possible false continuation. This is in addition to the fact that the termination locations based on QA present good agreement with white matter regions segmented from T_1_-weighted images (as shown in Figure 13), suggesting that QA can also delineate white matter region as the anatomy-based approach.

Another contribution of this paper is the generalized deterministic fiber tracking algorithm. This algorithm can make use of any voxel-based index (e.g. FA, GFA, or anatomical labels) or ODF-based index (e.g. QA) to conduct fiber filtering and define track termination. This algorithm is also a general version of deterministic fiber tracking because applying this algorithm to DTI will generate identical results as the FACT method. Furthermore, this algorithm can combine different interpolation methods to calculate trajectories. In this study, we used only trilinear interpolation, a method that has been applied to DTI tractography. It is possible to use a better interpolation strategy (e.g. spline) to improve the tracking results. We also ensured that this algorithm is reproducible and open to the public for peer examination. The detailed algorithm is documented in this paper, and the source codes are available online for public use (http://dsi-studio.labsolver.org).

The major limitation of this study is that we only used the arcuate fasciculus to evaluate tractography results, and it is still questionable whether the results are equally valid using other fiber pathways. This limitation is due to the practical considerations that not every neurosurgeon is experienced in all white matter structures and that the evaluation process is highly labor intensive. Importantly, there is no gold standard to define the exact anatomy of fiber tracks in the human brain, and the fiber microdissection used here represents an approximation method. A more comprehensive study can be done to examine the performance of our tracking method. Likewise, our in-vivo study did not investigate the effects of seeding strategy, step size, angular threshold, or interpolation method although they may affect the performance of a tracking algorithm. A more comprehensive study should be carried out to further investigate these effects. Similarly, a more complex phantom comprising different volume fractions and crossing angles is warranted to evaluate QA. Moreover, the grid and shell schemes were acquired with different echo times and scanning times. Therefore, the results do not necessarily suggest which scheme performed better. Last, the anatomy-aided tractography used in our study is only a simplified version, compared with other anatomy-based approaches [20,21], and we did not implement track rejection or continuation to fully exploit its power. It is possible that our tracking method can be further improved using anatomy prior to achieve better cortical localization for defining connectomics.

In conclusion, deterministic fiber tracking can be improved by using QA to filter noisy fibers and define track terminations. Our phantom study suggests that QA is less sensitive to the partial volume effect and may serve as a good index to define the extent of fiber pathways. Our *in vivo* study also confirms that the QA-aided tractography can achieve better accuracy than those aided by FA, GFA, and anatomy prior. Furthermore, the termination locations of the QA-aided tractography are in a good agreement with the cortical surface rendered by T_1_-weighted images. The improvement may facilitate further human connectome study that heavily relies on the accuracy of deterministic fiber tracking to define cortical connections. 
