# Correction: Deterministic Diffusion Fiber Tracking Improved by Quantitative Anisotropy

**DOI:** 10.1371/annotation/0f3b12de-8b8b-4dda-9ff4-835b8631e1dc

**Published:** 2014-01-02

**Authors:** Fang-Cheng Yeh, Timothy D. Verstynen, Yibao Wang, Juan C. Fernández-Miranda, Wen-Yih Isaac Tseng

In the Materials and Methods, the paragraph after Equation 3 contains several errors that were introduced during production. The third sentence of that paragraph should read "the nearby voxels include (32, 12, 16), (32, 12, 17), (32, 13, 16), (32, 13, 17), (33, 12, 16), (33, 12, 17), (33, 13, 16), and (33, 13, 17)."

In the fifth sentence of that paragraph, half of the equation is missing. Please see the full equation here: http://plosone.org/corrections/pone.0080713.e000.cn.tif

There are also errors in Table 1. Please refer to the corrected Table 1 here: http://plosone.org/corrections/pone.0080713.t001.cn.tif

